# Tetra­butyl­ammonium butyl­tetra­chlorido­stannate(IV)

**DOI:** 10.1107/S1600536813026172

**Published:** 2013-09-28

**Authors:** Tidiane Diop, Arie van der Lee, Libasse Diop

**Affiliations:** a Laboratoire de Chimie Minérale et Analytique, Département de Chimie, Faculté des Sciences et Techniques, Université Cheikh Anta Diop, Dakar, Senegal; b Institut Européen des Membranes, Université de Montpellier II, 34000, Montpellier, France

## Abstract

In the title compound, [N(C_4_H_9_)_4_][Sn(C_4_H_9_)Cl_4_], the Sn^IV^ atom of the stannate anion has a trigonal-bipyramidal coordination sphere by two Cl atoms and one butyl chain in the equatorial plane and by two Cl atoms in the apical positions. Two of the four butyl chains of the tetra­butyl­ammonium cation are partially disordered, each with refined site occupancies of 0.691 (6):0.309 (6). Weak C—H⋯Cl hydrogen-bonding inter­actions help to consolidate the crystal packing, as well as a short Cl⋯Cl inter­action of 3.295 (2) Å.

## Related literature

For general background to and applications of tin(IV) compounds, see: Evans & Karpel (1985 ▶); Davies *et al.* (2008 ▶). For related structures, see: Webster *et al.* (1976 ▶); Sow *et al.* (2010 ▶). For short Cl⋯Cl inter­actions in other chlorido­tin(IV) complexes, see: Brazeau *et al.* (2012 ▶); Cabon *et al.* (2010 ▶). For background to the weighting schemes used in the refinement, see: Prince (1982 ▶); Watkin (1994 ▶).
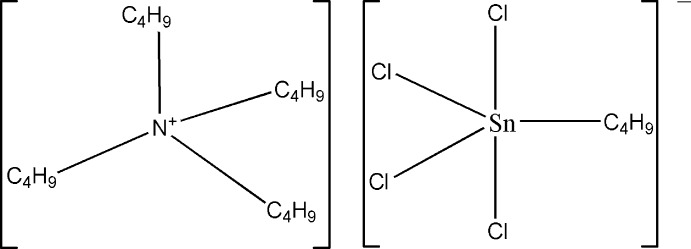


## Experimental

### 

#### Crystal data

(C_16_H_36_N)[Sn(C_4_H_9_)Cl_4_]
*M* *_r_* = 560.08
Triclinic, 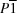

*a* = 11.6933 (5) Å
*b* = 11.7463 (5) Å
*c* = 12.2301 (6) Å
α = 114.236 (5)°
β = 101.680 (4)°
γ = 104.123 (4)°
*V* = 1395.80 (14) Å^3^
*Z* = 2
Mo *K*α radiation
μ = 1.30 mm^−1^
*T* = 175 K
0.45 × 0.40 × 0.15 mm

#### Data collection

Agilent Xcalibur (Sapphire3, Gemini) diffractometer
Absorption correction: multi-scan (*CrysAlis PRO*; Agilent, 2010 ▶) *T*_min_ = 0.803, *T*_max_ = 1.000
18571 measured reflections
6635 independent reflections
5438 reflections with *I* > 2σ(*I*)
*R*_int_ = 0.039

#### Refinement

*R*[*F* ^2^ > 2σ(*F* ^2^)] = 0.049
*wR*(*F* ^2^) = 0.088
*S* = 0.97
6628 reflections
254 parameters
142 restraints
H-atom parameters constrained
Δρ_max_ = 1.32 e Å^−3^
Δρ_min_ = −1.00 e Å^−3^

### 

Data collection: *CrysAlis PRO* (Agilent, 2010 ▶); cell refinement: *CrysAlis PRO*; data reduction: *CrysAlis PRO*; program(s) used to solve structure: *SUPERFLIP* (Palatinus & Chapuis, 2007 ▶); program(s) used to refine structure: *CRYSTALS* (Betteridge *et al.*, 2003 ▶); molecular graphics: *OLEX2* (Dolomanov *et al.*, 2009 ▶) and *VESTA* (Momma & Izumi, 2011 ▶); software used to prepare material for publication: *CRYSTALS*.

## Supplementary Material

Crystal structure: contains datablock(s) global, I. DOI: 10.1107/S1600536813026172/wm2766sup1.cif

Structure factors: contains datablock(s) I. DOI: 10.1107/S1600536813026172/wm2766Isup2.hkl

Additional supplementary materials:  crystallographic information; 3D view; checkCIF report

## Figures and Tables

**Table 1:** Selected bond lengths (Å)

Sn1—C6	2.129 (5)
Sn1—Cl2	2.3390 (12)
Sn1—Cl3	2.3494 (14)
Sn1—Cl4	2.4812 (14)
Sn1—Cl5	2.5051 (14)

**Table 2:** Hydrogen-bond geometry (Å, °)

*D*—H⋯*A*	*D*—H	H⋯*A*	*D*⋯*A*	*D*—H⋯*A*
C15—H152⋯Cl4	0.96	2.81	3.752 (6)	167
C19—H191⋯Cl5^i^	0.96	2.88	3.837 (5)	171
C19—H192⋯Cl5^ii^	0.96	2.90	3.830 (5)	164

Symmetry codes: (i) 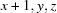; (ii) 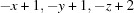.

**Fig. 1.**
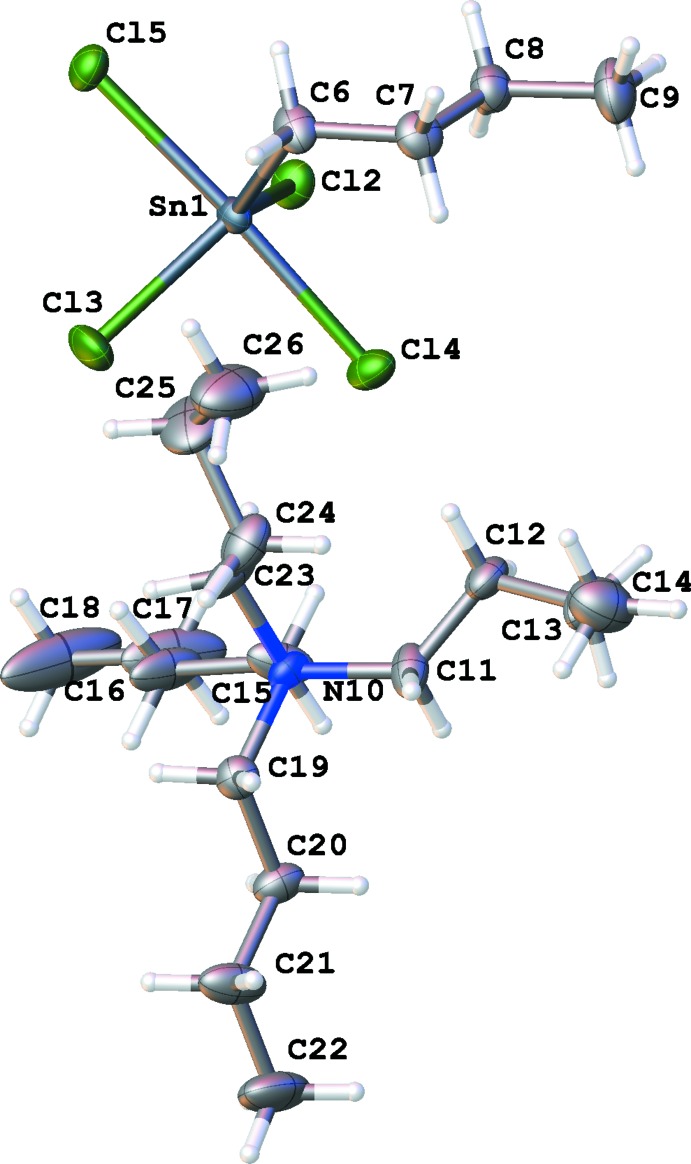
The title compound with displacement ellipsoids drawn at the 30% probability level. H atoms are shown as spheres of arbitary radius. Only the major component of the disordered part of the tetrabutylammonium cation is drawn for the sake of clarity.

**Fig. 2.**
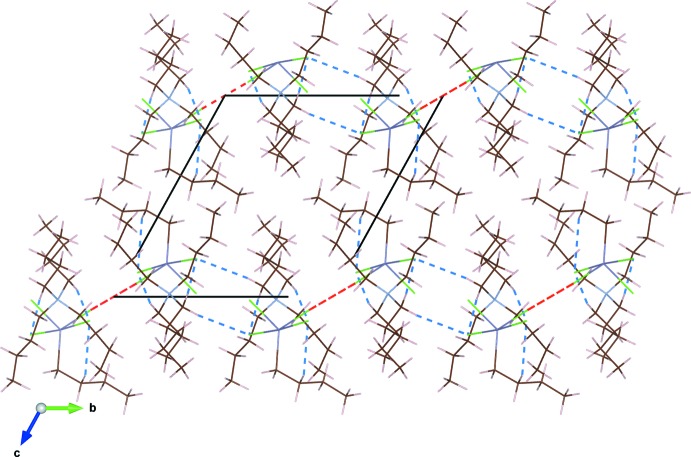
The packing of the structure viewed along the a axis in wireframe style. Intermolecular Cl···Cl interactions are drawn as dashed red lines and intermolecular C—H···Cl interactions as dashed blue lines.

## supplementary crystallographic information

### 1. Comment

The interest in new organotin(IV) derivatives is related to their applications in different fields: stabilization of polyvinyl chloride, treatments for glass surface, homogeneous catalysts, textile treatments and fungicidal properties (Evans & Karpel, 1985; Davies *et al.*, 2008).

The asymmetric unit of the title compound, (N(C_4_H_9_)_4_)[Sn(C_4_H_9_)Cl_4_], is illustrated in Fig. 1. It consists of a tetrabutylammonium cation and a tetrachloridobutylstannate(IV) anion. The Sn(IV) atom is five-coordinated in a distorted trigonal-bipyramidal arrangement with Cl4 and Cl5 in the apical positions. Major bond angle deviations with respect to the ideal trigonal-bipyramidal coordination geometry appear to be related to the different sizes of the butyl group and the Cl atoms. Thus in the equitorial plane, the C6—Sn1—Cl2 angle (125.04 (15)°) is larger than the Cl2—Sn1—Cl3 angle (112.46 (6)°). The angle Cl4—Sn1—Cl5 is 175.24 (6)°, indicating a slight deviation from linearity. The local geometry at the Sn(IV) position in the title compound is thus similar to that in (Ph_4_As)[MeSnCl_4_] (Webster *et al.*, 1976). The Sn—Cl distances, [2.3390 (12), 2.3494 (14), 2.4812 (14) and 2.5051 (14) Å], are slightly outside the range of the Sn—Cl distances [2.369 (4) and 2.400 (4) Å] observed in (*n*-Pr_2_NH_2_)[Sn(C_2_O_4_)Cl_4_] (Sow *et al.*, 2010). The C—N—C angles of the cation are close to 109°, in agreement with the expected *sp*^3^ hybridization.

The packing of the structure is shown in Fig. 2. A short intermolecular Cl2···Cl2 interaction of 3.295 (2) Å is found between two SnBuCl_4_^-^ anions related by an inversion center. Only two other non-disordered compounds containing chloridotin(IV) complexes have shorter Cl···Cl intermolecular interactions: 3.190 Å in [C_33_H_25_N_3_SnCl_4_]·CH_2_Cl_2_ (Brazeau *et al.*, 2012); 3.288 Å in *trans*-[PdCl(SnCl_3_(2-PyPPh_2_)_2_)] (Cabon *et al.*, 2010). In the title compound, cations and anions stack along the *a*-axis and are connected *via* weak C—H···Cl hydrogen-bonding interactions (Fig. 2). The stacks themselves are connected *via* weak Cl···Cl interactions and another C—H···Cl interaction as to form sheets parallel to the *ab* plane.

### 2. Experimental

Ethanolic solutions containing (NBu_4_)HSO_4_ (1.26 g, 4 mmol) and SnBuCl_3_ 2.25 g, 8 mmol) were mixed and stirred at room temperature for more than 1 h. After removing the precipitate, the filtrate was allowed to evaporate to give colourless crystals of the title compound. The idealized overall reaction is: (NBu_4_)HSO_4_ + 2 SnBuCl_3_→ (NBu_4_)[SnBuCl_4_] + SnBuCl_2_HSO_4_

### 3. Refinement

Three reflections, (0 1 1), (1 0 0) and (1 1 1), were obstructed by the beam stop and were omitted from the refinement. Disorder is observed for the dibutyl ammonium ion. Two of the four butyl chains of the tetrabutylammonium cation are partially disordered and were refined with site occupancies of 0.691 (6):0.309 (6) for each chain. All equivalent disordered moieties were restrained to have similar geometries. The displacement parameters of disordered atoms C12, C121, C24 and C241 of the tetrabutylammonium cation were restrained to be approximately isotropic. The H atoms were initially refined with soft restraints on the bond lengths and angles to regularize their geometry (C—H in the range 0.93–0.98, Å) and *U*_iso_(H) (in the range 1.2–1.5 times *U*_eq_ of the parent atom), after which the positions were refined with riding constraints.

### Crystal data

**Table:**

(C_16_H_36_N)[Sn(C_4_H_9_)Cl_4_]	*Z* = 2
*M**_r_* = 560.08	*F*(000) = 580.000
Triclinic, *P*1	*D*_x_ = 1.333 Mg m^−^^3^
Hall symbol: -P 1	Mo *K*α radiation, λ = 0.71073 Å
*a* = 11.6933 (5) Å	Cell parameters from 5275 reflections
*b* = 11.7463 (5) Å	θ = 2.0–28.2°
*c* = 12.2301 (6) Å	µ = 1.30 mm^−^^1^
α = 114.236 (5)°	*T* = 175 K
β = 101.680 (4)°	Plate, colourless
γ = 104.123 (4)°	0.45 × 0.40 × 0.15 mm
*V* = 1395.80 (14) Å^3^	

### Data collection

**Table:**

Agilent Xcalibur (Sapphire3, Gemini) diffractometer	6635 independent reflections
Radiation source: Enhance (Mo) X-ray Source	5438 reflections with *I* > 2σ(*I*)
Graphite monochromator	*R*_int_ = 0.039
Detector resolution: 16.0143 pixels mm^-1^	θ_max_ = 29.2°, θ_min_ = 1.9°
ω scans	*h* = −15→16
Absorption correction: multi-scan (*CrysAlis PRO*; Agilent, 2010)	*k* = −15→15
*T*_min_ = 0.803, *T*_max_ = 1.000	*l* = −16→16
18571 measured reflections	

### Refinement

**Table:**

Refinement on *F*^2^	Primary atom site location: iterative
Least-squares matrix: full	Hydrogen site location: difference Fourier map
*R*[*F*^2^ > 2σ(*F*^2^)] = 0.049	H-atom parameters constrained
*wR*(*F*^2^) = 0.088	Method, part 1, Chebychev polynomial, (Watkin, 1994, *P*rince, 1982) [*w*eight] = 1.0/[A_0_*T_0_(x) + A_1_*T_1_(x) ··· + A_n-1_]*T_n-1_(x)] where A_i_ are the Chebychev coefficients listed belo*w* and x = *F* /*F*max Method = Robust Weighting (*P*rince, 1982) W = [*w*eight] * [1-(delta*F*/6*sigma*F*)^2^]^2^ A_i_ are: 0.178E + 04 0.238E + 04 0.133E + 04 350. 47.8
*S* = 0.97	(Δ/σ)_max_ = 0.001
6628 reflections	Δρ_max_ = 1.32 e Å^−^^3^
254 parameters	Δρ_min_ = −1.00 e Å^−^^3^
142 restraints	

### Special details

**Table:**

Experimental. The crystal was placed in the cold stream of an Oxford Cryosystems open-flow nitrogen cryostat (Cosier & Glazer, 1986) with a nominal stability of 0.1 K.Cosier, J. & Glazer, A·*M*., 1986. J. Appl. Cryst. 105–107.

### Fractional atomic coordinates and isotropic or equivalent isotropic displacement parameters (Å^2^)

**Table:**

	*x*	*y*	*z*	*U*_iso_*/*U*_eq_	Occ. (<1)
Sn1	0.19913 (3)	0.15984 (3)	0.83567 (3)	0.0345	
Cl2	0.07714 (13)	0.07249 (13)	0.93279 (13)	0.0501	
Cl3	0.35953 (15)	0.36814 (14)	0.98257 (16)	0.0696	
Cl4	0.34432 (13)	0.04890 (15)	0.87972 (15)	0.0554	
Cl5	0.05658 (13)	0.28254 (14)	0.81078 (14)	0.0522	
C6	0.1661 (6)	0.0700 (5)	0.6359 (5)	0.0528	
C7	0.1672 (6)	−0.0706 (6)	0.5700 (5)	0.0542	
C8	0.0680 (6)	−0.1742 (5)	0.5766 (5)	0.0541	
C9	0.0661 (8)	−0.3156 (6)	0.5062 (6)	0.0838	
H91	0.0041	−0.3756	0.5178	0.1202*	
H93	0.0471	−0.3436	0.4171	0.1200*	
H92	0.1472	−0.3155	0.5410	0.1201*	
H82	−0.0138	−0.1753	0.5366	0.0644*	
H81	0.0801	−0.1504	0.6647	0.0641*	
H72	0.1551	−0.0971	0.4805	0.0641*	
H71	0.2490	−0.0689	0.6100	0.0640*	
H61	0.2285	0.1287	0.6217	0.0612*	
H62	0.0829	0.0638	0.5954	0.0613*	
N10	0.7478 (4)	0.2479 (4)	0.9853 (4)	0.0392	
C11	0.7451 (6)	0.1082 (5)	0.9094 (5)	0.0586	
C12	0.6356 (7)	−0.0102 (7)	0.8184 (7)	0.0540	0.691 (6)
C13	0.6825 (7)	−0.1149 (7)	0.7370 (5)	0.0814	
C14	0.7350 (7)	−0.1247 (9)	0.6359 (7)	0.1001	
H142	0.7377	−0.2109	0.5927	0.1750*	
H141	0.8180	−0.0586	0.6779	0.1751*	
H143	0.6843	−0.1054	0.5796	0.1750*	
C15	0.6838 (5)	0.2475 (7)	1.0813 (5)	0.0657	
C16	0.6844 (7)	0.3808 (9)	1.1736 (6)	0.0926	
C17	0.6118 (8)	0.3602 (12)	1.2581 (7)	0.1457	
C18	0.6068 (11)	0.4897 (14)	1.3521 (8)	0.2270	
H182	0.5562	0.4628	1.4005	0.3500*	
H183	0.6856	0.5485	1.4059	0.3500*	
H181	0.5606	0.5205	1.3057	0.3500*	
H172	0.6543	0.3240	1.3055	0.1789*	
H171	0.5285	0.2957	1.2041	0.1789*	
H161	0.7696	0.4436	1.2251	0.1170*	
H162	0.6441	0.4184	1.1265	0.1170*	
H151	0.7288	0.2166	1.1315	0.0782*	
H152	0.5991	0.1855	1.0344	0.0781*	
C19	0.8824 (5)	0.3421 (5)	1.0513 (5)	0.0448	
C20	0.9606 (5)	0.3237 (6)	1.1534 (5)	0.0526	
C21	1.0970 (6)	0.4119 (8)	1.1979 (6)	0.0762	
C22	1.1793 (7)	0.3939 (9)	1.2977 (7)	0.1068	
H221	1.2653	0.4435	1.3186	0.1650*	
H223	1.1678	0.2998	1.2639	0.1650*	
H222	1.1575	0.4216	1.3733	0.1649*	
H212	1.1254	0.3905	1.1250	0.0922*	
H211	1.1033	0.5062	1.2359	0.0922*	
H201	0.9538	0.2311	1.1178	0.0639*	
H202	0.9289	0.3467	1.2239	0.0643*	
H191	0.9208	0.3319	0.9868	0.0541*	
H192	0.8825	0.4319	1.0916	0.0543*	
C23	0.6808 (7)	0.2893 (7)	0.9006 (5)	0.0727	
C24	0.6979 (9)	0.2669 (10)	0.7805 (8)	0.0703	0.691 (6)
C25	0.5893 (8)	0.2945 (9)	0.7078 (7)	0.1069	
C26	0.6013 (9)	0.2481 (10)	0.5815 (7)	0.1269	
H262	0.5538	0.2733	0.5292	0.2270*	
H261	0.6884	0.2824	0.5946	0.2270*	
H263	0.5722	0.1505	0.5403	0.2270*	
H241	0.7797	0.3319	0.8013	0.1187*	0.691 (6)
H242	0.6970	0.1809	0.7362	0.1187*	0.691 (6)
C121	0.7471 (16)	0.0365 (11)	0.7809 (10)	0.0577	0.309 (6)
C241	0.5625 (11)	0.2512 (19)	0.8044 (14)	0.0699	0.309 (6)
H121	0.5856	0.0133	0.7658	0.0975*	0.691 (6)
H122	0.5903	−0.0407	0.8624	0.0975*	0.691 (6)
H1211	0.8314	0.0601	0.7850	0.0600*	0.309 (6)
H1212	0.7041	0.0672	0.7270	0.0600*	0.309 (6)
H2411	0.5153	0.1542	0.7646	0.0600*	0.309 (6)
H2412	0.5081	0.2920	0.8470	0.0600*	0.309 (6)
H111	0.8000	0.1167	0.8633	0.0751*	0.691 (6)
H112	0.7805	0.0864	0.9717	0.0751*	0.691 (6)
H113	0.8166	0.1070	0.9614	0.0751*	0.309 (6)
H114	0.6711	0.0516	0.9086	0.0751*	0.309 (6)
H231	0.7030	0.3834	0.9501	0.0874*	0.691 (6)
H232	0.5937	0.2452	0.8808	0.0874*	0.691 (6)
H233	0.7374	0.2994	0.8564	0.0874*	0.309 (6)
H234	0.6878	0.3753	0.9619	0.0874*	0.309 (6)
H251	0.6031	0.3875	0.7471	0.1274*	0.691 (6)
H252	0.5094	0.2458	0.7040	0.1274*	0.691 (6)
H253	0.6697	0.3632	0.7582	0.1274*	0.309 (6)
H254	0.5284	0.3339	0.6994	0.1274*	0.309 (6)
H131	0.7456	−0.1166	0.7983	0.0974*	0.691 (6)
H132	0.6119	−0.1964	0.6969	0.0974*	0.691 (6)
H133	0.7157	−0.1461	0.7920	0.0974*	0.309 (6)
H134	0.5932	−0.1484	0.7118	0.0974*	0.309 (6)

### Atomic displacement parameters (Å^2^)

**Table:**

	*U*^11^	*U*^22^	*U*^33^	*U*^12^	*U*^13^	*U*^23^
Sn1	0.03730 (17)	0.03018 (15)	0.03305 (16)	0.00540 (12)	0.00836 (12)	0.01820 (12)
Cl2	0.0569 (8)	0.0525 (7)	0.0618 (8)	0.0205 (6)	0.0315 (7)	0.0408 (7)
Cl3	0.0570 (9)	0.0399 (7)	0.0734 (10)	−0.0079 (6)	−0.0061 (8)	0.0208 (7)
Cl4	0.0447 (7)	0.0638 (9)	0.0735 (10)	0.0213 (7)	0.0157 (7)	0.0484 (8)
Cl5	0.0568 (8)	0.0470 (7)	0.0678 (9)	0.0253 (6)	0.0236 (7)	0.0365 (7)
C6	0.074 (4)	0.048 (3)	0.038 (3)	0.015 (3)	0.018 (3)	0.027 (2)
C7	0.066 (4)	0.058 (3)	0.039 (3)	0.022 (3)	0.023 (3)	0.021 (3)
C8	0.067 (4)	0.045 (3)	0.044 (3)	0.019 (3)	0.017 (3)	0.017 (3)
C9	0.125 (7)	0.047 (4)	0.066 (4)	0.030 (4)	0.030 (4)	0.017 (3)
N10	0.042 (2)	0.049 (2)	0.041 (2)	0.0283 (18)	0.0206 (18)	0.0260 (17)
C11	0.100 (4)	0.042 (2)	0.039 (3)	0.031 (2)	0.021 (2)	0.0226 (18)
C12	0.065 (4)	0.058 (3)	0.050 (4)	0.039 (2)	0.016 (3)	0.029 (3)
C13	0.116 (5)	0.071 (3)	0.039 (3)	0.061 (4)	0.006 (3)	0.005 (2)
C14	0.082 (5)	0.106 (6)	0.078 (5)	0.027 (5)	0.018 (4)	0.024 (4)
C15	0.036 (3)	0.106 (5)	0.039 (3)	0.015 (3)	0.020 (2)	0.023 (3)
C16	0.080 (5)	0.151 (7)	0.042 (3)	0.086 (5)	0.023 (3)	0.019 (4)
C17	0.102 (7)	0.294 (15)	0.051 (4)	0.122 (9)	0.048 (5)	0.055 (7)
C18	0.222 (13)	0.43 (2)	0.053 (5)	0.270 (15)	0.051 (7)	0.047 (9)
C19	0.048 (3)	0.050 (3)	0.046 (3)	0.017 (2)	0.021 (2)	0.028 (2)
C20	0.051 (3)	0.075 (4)	0.049 (3)	0.036 (3)	0.022 (3)	0.035 (3)
C21	0.046 (3)	0.115 (6)	0.048 (3)	0.036 (4)	0.017 (3)	0.019 (4)
C22	0.064 (5)	0.139 (8)	0.088 (6)	0.060 (5)	0.003 (4)	0.028 (5)
C23	0.102 (4)	0.094 (4)	0.043 (3)	0.079 (4)	0.019 (3)	0.030 (3)
C24	0.096 (5)	0.079 (5)	0.079 (4)	0.053 (5)	0.047 (4)	0.056 (5)
C25	0.148 (6)	0.114 (6)	0.074 (4)	0.059 (5)	0.009 (4)	0.066 (4)
C26	0.131 (7)	0.154 (8)	0.068 (4)	0.077 (6)	0.004 (4)	0.029 (5)
C121	0.072 (7)	0.092 (4)	0.042 (4)	0.059 (6)	0.024 (5)	0.043 (4)
C241	0.063 (5)	0.069 (8)	0.102 (7)	0.042 (6)	0.017 (4)	0.059 (6)

### Geometric parameters (Å, º)

**Table:**

Sn1—C6	2.129 (5)	C17—H172	0.975
Sn1—Cl2	2.3390 (12)	C17—H171	0.963
Sn1—Cl3	2.3494 (14)	C18—H182	1.003
Sn1—Cl4	2.4812 (14)	C18—H183	0.910
Sn1—Cl5	2.5051 (14)	C18—H181	0.940
C6—C7	1.516 (7)	C19—C20	1.516 (7)
C6—H61	0.971	C19—H191	0.964
C6—H62	0.969	C19—H192	0.963
C7—C8	1.505 (7)	C20—C21	1.515 (8)
C7—H72	0.975	C20—H201	0.966
C7—H71	0.974	C20—H202	0.970
C8—C9	1.517 (7)	C21—C22	1.515 (9)
C8—H82	0.976	C21—H212	0.972
C8—H81	0.965	C21—H211	0.986
C9—H91	0.957	C22—H221	0.951
C9—H93	0.957	C22—H223	0.970
C9—H92	0.957	C22—H222	0.956
N10—C11	1.505 (6)	C23—C24	1.446 (7)
N10—C15	1.517 (6)	C23—C241	1.450 (9)
N10—C19	1.501 (6)	C23—H231	0.950
N10—C23	1.483 (6)	C23—H232	0.950
C11—C12	1.447 (7)	C23—H233	0.950
C11—C121	1.455 (9)	C23—H234	0.950
C11—H111	0.950	C24—C25	1.578 (7)
C11—H112	0.950	C24—H241	0.974
C11—H113	0.950	C24—H242	0.925
C11—H114	0.950	C24—C241	1.648 (16)
C12—C13	1.532 (7)	C24—H233	0.818
C12—C121	1.520 (16)	C25—C26	1.462 (7)
C12—H121	0.950	C25—C241	1.523 (9)
C12—H122	0.939	C25—H251	0.950
C12—H114	0.963	C25—H252	0.950
C13—C14	1.465 (10)	C25—H253	0.950
C13—C121	1.556 (9)	C25—H254	0.950
C13—H131	0.950	C26—H262	0.946
C13—H132	0.950	C26—H261	0.952
C13—H133	0.950	C26—H263	0.975
C13—H134	0.954	C121—H1211	0.941
C14—H142	0.943	C121—H1212	0.983
C14—H141	0.954	C121—H111	0.985
C14—H143	0.952	C241—H2411	0.984
C15—C16	1.505 (9)	C241—H2412	0.992
C15—H151	0.964	C241—H232	0.969
C15—H152	0.957	C241—H252	1.227
C16—C17	1.519 (10)	H112—H113	0.499
C16—H161	0.969	H131—H133	0.395
C16—H162	0.975	H132—H134	0.626
C17—C18	1.508 (13)		
			
Cl2—Sn1—Cl3	112.46 (6)	N10—C19—H192	107.4
Cl2—Sn1—Cl4	88.66 (5)	C20—C19—H192	107.4
Cl3—Sn1—Cl4	88.58 (6)	H191—C19—H192	109.4
Cl2—Sn1—Cl5	88.99 (5)	C19—C20—C21	109.9 (5)
Cl3—Sn1—Cl5	88.51 (6)	C19—C20—H201	109.0
Cl4—Sn1—Cl5	175.24 (6)	C21—C20—H201	108.7
Cl2—Sn1—C6	125.04 (15)	C19—C20—H202	108.7
Cl3—Sn1—C6	122.42 (15)	C21—C20—H202	110.6
Cl4—Sn1—C6	95.13 (16)	H201—C20—H202	109.9
Cl5—Sn1—C6	89.61 (16)	C20—C21—C22	111.7 (6)
Sn1—C6—C7	117.6 (3)	C20—C21—H212	109.2
Sn1—C6—H61	107.8	C22—C21—H212	109.8
C7—C6—H61	108.6	C20—C21—H211	108.1
Sn1—C6—H62	106.8	C22—C21—H211	108.6
C7—C6—H62	105.9	H212—C21—H211	109.4
H61—C6—H62	110.0	C21—C22—H221	110.4
C6—C7—C8	114.0 (5)	C21—C22—H223	109.3
C6—C7—H72	108.7	H221—C22—H223	108.2
C8—C7—H72	108.6	C21—C22—H222	111.2
C6—C7—H71	108.3	H221—C22—H222	109.5
C8—C7—H71	108.4	H223—C22—H222	108.1
H72—C7—H71	108.8	N10—C23—C24	122.5 (5)
C7—C8—C9	113.8 (5)	N10—C23—C241	143.6 (8)
C7—C8—H82	108.0	C24—C23—C241	69.3 (7)
C9—C8—H82	106.9	N10—C23—H231	106.0
C7—C8—H81	109.5	C24—C23—H231	106.1
C9—C8—H81	108.9	C241—C23—H231	102.1
H82—C8—H81	109.6	N10—C23—H232	106.2
C8—C9—H91	109.6	C24—C23—H232	106.2
C8—C9—H93	109.9	H231—C23—H232	109.5
H91—C9—H93	109.6	N10—C23—H233	100.4
C8—C9—H92	108.2	C241—C23—H233	100.8
H91—C9—H92	109.5	H231—C23—H233	93.0
H93—C9—H92	109.9	H232—C23—H233	138.2
C11—N10—C15	107.9 (4)	N10—C23—H234	100.2
C11—N10—C19	108.4 (4)	C24—C23—H234	121.2
C15—N10—C19	111.0 (4)	C241—C23—H234	100.2
C11—N10—C23	110.7 (4)	H232—C23—H234	97.0
C15—N10—C23	109.5 (4)	H233—C23—H234	109.5
C19—N10—C23	109.4 (4)	C23—C24—C25	105.9 (6)
N10—C11—C12	126.8 (5)	C23—C24—H241	106.6
N10—C11—C121	131.7 (6)	C25—C24—H241	110.9
C12—C11—C121	63.1 (7)	C23—C24—H242	110.2
N10—C11—H111	105.1	C25—C24—H242	113.5
C12—C11—H111	105.3	H241—C24—H242	109.5
N10—C11—H112	104.7	C23—C24—C241	55.5 (5)
C12—C11—H112	104.9	C25—C24—C241	56.3 (4)
C121—C11—H112	118.5	H241—C24—C241	143.5
H111—C11—H112	109.5	H242—C24—C241	106.8
N10—C11—H113	103.1	C25—C24—H233	132.3
C12—C11—H113	124.0	H241—C24—H233	71.0
C121—C11—H113	103.5	H242—C24—H233	109.8
H111—C11—H113	80.9	C241—C24—H233	92.9
N10—C11—H114	104.3	C24—C25—C26	101.9 (7)
C121—C11—H114	103.7	C24—C25—C241	64.2 (6)
H111—C11—H114	145.4	C26—C25—C241	144.2 (11)
H112—C11—H114	79.8	C24—C25—H251	110.9
H113—C11—H114	109.5	C26—C25—H251	110.8
C11—C12—C13	107.2 (6)	C241—C25—H251	104.9
C11—C12—C121	58.7 (5)	C24—C25—H252	111.8
C13—C12—C121	61.3 (5)	C26—C25—H252	111.9
C11—C12—H121	108.2	C241—C25—H252	53.6
C13—C12—H121	109.7	H251—C25—H252	109.5
C121—C12—H121	90.6	C24—C25—H253	59.3
C11—C12—H122	109.3	C26—C25—H253	99.8
C13—C12—H122	112.0	C241—C25—H253	99.8
C121—C12—H122	158.8	H251—C25—H253	56.4
H121—C12—H122	110.4	H252—C25—H253	148.4
C13—C12—H114	128.6	C24—C25—H254	156.4
C121—C12—H114	98.6	C26—C25—H254	100.5
H121—C12—H114	117.7	C241—C25—H254	100.7
H122—C12—H114	69.2	H251—C25—H254	53.2
C12—C13—C14	131.8 (7)	H252—C25—H254	65.8
C12—C13—C121	59.0 (6)	H253—C25—H254	109.5
C14—C13—C121	79.8 (6)	C25—C26—H262	113.7
C12—C13—H131	103.6	C25—C26—H261	106.8
C14—C13—H131	103.7	H262—C26—H261	111.5
C121—C13—H131	95.0	C25—C26—H263	107.0
C12—C13—H132	103.7	H262—C26—H263	108.9
C14—C13—H132	103.5	H261—C26—H263	108.6
C121—C13—H132	153.4	C13—C121—C12	59.8 (5)
H131—C13—H132	109.5	C13—C121—C11	105.6 (7)
C12—C13—H133	104.1	C12—C121—C11	58.2 (5)
C14—C13—H133	116.7	C13—C121—H1211	113.1
C121—C13—H133	116.3	C12—C121—H1211	156.6
H132—C13—H133	86.3	C11—C121—H1211	107.9
C12—C13—H134	68.6	C13—C121—H1212	113.8
C14—C13—H134	116.4	C12—C121—H1212	95.4
C121—C13—H134	116.2	C11—C121—H1212	108.9
H131—C13—H134	132.0	H1211—C121—H1212	107.4
H133—C13—H134	109.1	C13—C121—H111	134.7
C13—C14—H142	110.2	C12—C121—H111	98.5
C13—C14—H141	105.8	H1211—C121—H111	70.3
H142—C14—H141	110.2	H1212—C121—H111	107.1
C13—C14—H143	108.0	C25—C241—C23	108.6 (8)
H142—C14—H143	111.5	C25—C241—C24	59.5 (5)
H141—C14—H143	111.0	C23—C241—C24	55.2 (5)
N10—C15—C16	116.4 (6)	C25—C241—H2411	112.5
N10—C15—H151	106.1	C23—C241—H2411	110.2
C16—C15—H151	106.8	C24—C241—H2411	106.2
N10—C15—H152	107.5	C25—C241—H2412	113.0
C16—C15—H152	109.5	C23—C241—H2412	108.9
H151—C15—H152	110.4	C24—C241—H2412	149.9
C15—C16—C17	109.1 (8)	H2411—C241—H2412	103.4
C15—C16—H161	110.7	C25—C241—H232	149.0
C17—C16—H161	110.4	C24—C241—H232	91.9
C15—C16—H162	109.5	H2411—C241—H232	85.3
C17—C16—H162	109.1	H2412—C241—H232	85.2
H161—C16—H162	108.0	C23—C241—H252	147.1
C16—C17—C18	112.1 (11)	C24—C241—H252	94.0
C16—C17—H172	107.9	H2411—C241—H252	87.8
C18—C17—H172	108.6	H2412—C241—H252	92.4
C16—C17—H171	108.2	H232—C241—H252	171.9
C18—C17—H171	110.7	C121—H111—C11	97.6
H172—C17—H171	109.2	C11—H112—H113	74.8
C17—C18—H182	104.3	C11—H113—H112	74.8
C17—C18—H183	110.3	C12—H114—C11	98.3
H182—C18—H183	110.4	C241—H232—C23	98.2
C17—C18—H181	108.0	C23—H233—C24	109.5
H182—C18—H181	107.6	C241—H252—C25	87.8
H183—C18—H181	115.7	C13—H131—H133	78.0
N10—C19—C20	116.1 (4)	C13—H132—H134	71.2
N10—C19—H191	107.6	C13—H133—H131	78.0
C20—C19—H191	108.9	H132—H134—C13	70.5

### Hydrogen-bond geometry (Å, º)

**Table:**

*D*—H···*A*	*D*—H	H···*A*	*D*···*A*	*D*—H···*A*
C15—H152···Cl4	0.96	2.81	3.752 (6)	167
C19—H191···Cl5^i^	0.96	2.88	3.837 (5)	171
C19—H192···Cl5^ii^	0.96	2.90	3.830 (5)	164

Symmetry codes: (i) *x*+1, *y*, *z*; (ii) −*x*+1, −*y*+1, −*z*+2.
